# Explanted Skull Flaps after Decompressive Hemicraniectomy Demonstrate Relevant Bone Avitality-Is Their Reimplantation Worth the Risk?

**DOI:** 10.3390/brainsci13091277

**Published:** 2023-09-01

**Authors:** Konstantinos Gousias, Ingo Stricker, Annika Hoyer, Theocharis Theocharous, Csilla Rompf, Arthur B. Pranada, Andrea Tannapfel, Rachit Agrawal, Iris Tischoff

**Affiliations:** 1 Department of Neurosurgery, Academic Hospital of University of Muenster, St. Marien Hospital Luenen, 44532 Luenen, Germany; drtheocharoust@gmail.com (T.T.); smarterachitag@gmail.com (R.A.); 2 Medical School, Westfaelische Wilhelms University of Muenster, 48149 Muenster, Germany; 3 Medical School, University of Nicosia, Nicosia 2408, Cyprus; 4 Institute of Pathology, Ruhr University Bochum, 44789 Bochum, Germany; ingo.stricker@pathologie-bochum.de (I.S.); andrea.tannapfel@pathologie-bochum.de (A.T.); iris.tischoff@rub.de (I.T.); 5 Biostatistics and Medical Biometry, Medical School OWL, Bielefeld University, 33615 Bielefeld, Germany; annika.hoyer@uni-bielefeld.de; 6 MVZ Dr. Eberhard & Partner Dortmund, 44137 Dortmund, Germany; rompf@labmed.de (C.R.); apranada@labmed.de (A.B.P.)

**Keywords:** decompressive hemicraniectomy, skull bone flaps, storage, skull bone avitality, bone flap resorption

## Abstract

Background: Reimplantations of autologous skull flaps after decompressive hemicraniectomies (DHs) are associated with high rates of postoperative bone flap resorption (BFR). We histologically assessed the cell viability of explanted bone flaps in certain periods of time after DH, in order to conclude whether precursors of BRF may be developed during their storage. Methods: Skull bone flaps explanted during a DH between 2019 and 2020 were stored in a freezer at either −23 °C or −80 °C. After their thawing process, the skulls were collected. Parameters of bone metabolism, namely PTH1 and OPG, were analyzed via immunohistochemistry. H&E stain was used to assess the degree of avital bone tissue, whereas the repeated assays were performed after 6 months. Results: A total of 17 stored skull flaps (8 at −23 °C; 9 at −80 °C) were analyzed. The duration of cryopreservation varied between 2 and 17 months. A relevant degree of bone avitality was observed in all skull flaps, which significantly increased at the repeated evaluation after 6 months (*p* < 0.001). Preservation at −23 °C (*p* = 0.006) as well as longer storage times (*p* < 0.001) were identified as prognostic factors for higher rates of bone avitality in a linear mixed regression model. Conclusions: Our novel finding shows a clear benefit from storage at −80° C, which should be carefully considered for the future management and storage of explanted skull flaps. Our analysis also further revealed a significant degree of bone avitality, a potential precursor of BFR, in skull flaps stored for several weeks. To this end, we should reconsider whether the reimplantation of autologous skull flaps instead of synthetic skull flaps is still justified.

## 1. Introduction

Cranioplasty is defined as the reimplantation of either previously removed autologous skull flaps after a decompressive hemicraniectomy (DH) or implantation of a synthetic flap (SF) and is performed after regress of the malignant brain swelling at an interval of several weeks to months following DH [1,2]. Although the surgical procedure is regarded as one of the simplest in neurosurgery, it is associated with an unusually high rate of early and/or secondary postoperative complications [1,3,4,5,6,7]. Overall cranioplasty related complications are significantly higher after the reimplantation of an autologous bone flap (ABF) compared to an SF, mostly because of the development of bone flap resorption (BFR), which is only seen after cranioplasty with ABFs [6,8]. BFR, which is also called aseptic bone necrosis or osteonecrosis, has been long recognized as the most common ABF-specific cranioplasty complication with an estimated pooled incidence of 15% to almost every second patient, according to recently published clinical systematic reviews [3,4,6,8]. Further specific volumetric studies on ABFs identified much higher rates of BFR, namely from 77 to 90% [9,10]. Spake et al. reported a rate of 82% for clinically relevant BRF depicted on cranial CT among their cohort, whereas BRF progressed over the years in a linear and continuous manner [11]. 

To date, several factors have been associated with a higher probability of postoperative BFR, namely younger age [1,3,10,12,13], reimplantation of multi-fragmented skull flaps after traumatic brain injury [1,3,4,5,12,13], multiple skull operations [3,8,12] or the longer duration of surgical procedures [3,12]. 

In order to identify further potential sources of secondary aseptic bone necrosis after ABF cranioplasty, in particular to answer the question whether the process of necrosis begins directly after the DH and is ongoing during the storage of the skull flaps and whether the temperature (−23 °C vs. −80 °C) and duration of storage may influence this procedure, we conducted our clinical trial DRKS00023283 (www.drks.de, accessed on 26 October 2020). In this regard, we carried out a histological analysis of the disparately cryopreserved skull flaps and assessed via H&E staining the degree of bone avitality, a precursor of aseptic bone necrosis, in two different periods of time after DH, namely at the initial index time and after 6 months. Further parameters of bone metabolism, namely parathyroid hormone 1 receptor (PTH1) and osteoprotegerin (OPG) were analyzed via immunohistochemistry.

## 2. Methods

### 2.1. Patients

The clinical trial DRKS00023283 (http://www.drks.de, accessed on 26 October 2020). aimed to clarify whether different patterns of storage of explanted skull flaps after DH, in particular storage at different degrees of °C, may prognosticate late complications after cranioplasty, among others infection and aseptic bone necrosis of the skull flap implants. To this end, we analyzed explanted skull flaps of only deceased adult patients, who underwent a DH for an acute malignant brain swelling in our department between June 2019 and October 2020. The trial was approved by the local ethics committee of the University of Muenster (ethic votum: 2020-340-f-S). Informed consent for inclusion in the research and publication was obtained by their legal representatives.

In our analysis, we included explanted skull flaps of 17 deceased patients; 8 skulls were preserved at −23 °C (group A) and 9 at −80 °C (group B). The specific parameters of demographics, surgical procedure and storage, i.e., age (years), sex (m/w), cause of malignant brain swelling (stroke vs. severe traumatic brain injury (sTBI)), additional skull fracture (yes/no), infection prior to DH (yes/no), duration of DH (minutes), duration (months) and temperature of storage (23 °C vs. −80 °C) were included as potential prognostic factors in our statistical analysis. 

### 2.2. Material Collection, Patterns of Storage, Histological and Statistical Analysis

The skull bone flaps were explanted during a DH for a vascular disease, like infarct of arteria cerebri media (ACM) or sTBI between June 2019 and October 2020. After sterile packaging in triple plastic bags, the flaps were stored in a freezer at a temperature of either −23 °C (DHs between June and November 2019) or −80 °C (DHs between December 2019 and October 2020). The procedures of collecting and thawing the bone fragments (cortex and cancellous bone) were conducted as previously described [14]. 

**Figure 1:**
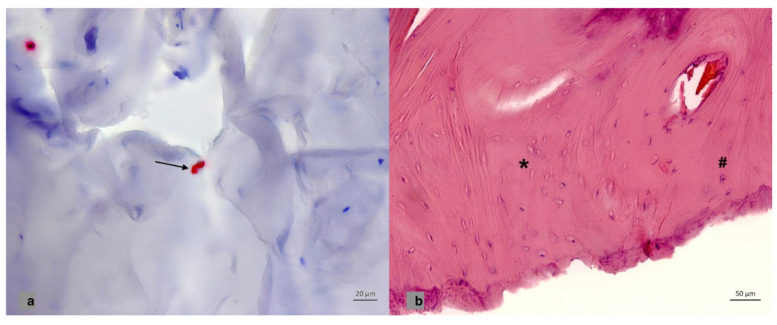
(**a**) Immunohistochemical staining in the cell membrane for PTH1R in bone fragments. Black arrow shows the expression for PTH1R in the cell membrane. (**b**) H&E staining of a bone fragment. The hashtag represents the normal osteocytes and the asterisk shows the empty osteocytic lacunae as a histological sign for osteonecrosis.

At least five centrally located bone fragments per skull flap, sized approximately 0.5 × 0.5 cm, were collected in sterile tubes filled with formaldehyde solution. Formalin-fixed paraffin-embedded (FFPE) specimens were decalcificated and briefly, 1–2 micron-thin sections were cut and stained with H&E according to routine protocols. Immunostaining of PTH1R and OPG was performed using a Leica BOND-MAX autostainer and a Bond Polymer Refine Red Detection system (Leica Microsystems, Wetzlar, Germany) according to the manufacturer’s specifications. PTH1R and OPG polyclonal antibodies were used according to the manufacturer’s protocols (PTH1R: Catalog PA1-20597, Invitrogen, dilution 1:200; OPG: bs-0431R, BIOSS). The immunoreactivity in the membrane for PTH1R and in cytoplasm for OPG was counted. The number of positive cells (osteoclasts, osteoblasts) in relation to all identified cells was documented. A Zeiss Light microscope (Zeiss Axio Imager.D2) was used for evaluation of PTH1R and OPG expression and H&E slides (Figure 1). Additionally, H&E slides were scanned via Light Microscope (Zeiss Axio Imager, Zeiss, Bochum, Germany. D2, Camera Zeiss Axiocam 506 color, Photography Zen2pro at 10–50 times magnification), whereas the degree of vitality in scanned H&E-stained images was calculated via Image J software, Version 1.53t. Avital tissue was defined as loss of osteocytes with empty osteocytic lacunae (Figure 1b). Empty osteocytic lacunae are a well-known histological sign for osteonecrosis and were detected and counted. Other known histological features for ischemia like ghosting in the fatty and haemopoietic marrow or proliferation of small vessels were only focally identified and not documented.

Wilcoxon tests were applied for comparisons between the group with bone flaps stored at −23 °C and the group with bone flaps stored at −80 °C. Log-linear mixed regression models with random intercept were used to account for repeated measures, i.e., expression of avital tissue of the same bone flaps, and to identify potential prognostic factors of bone avitality. According to this, the temperature of storage and time point of assessing avital areas were included as covariates. *p*-values < 0.05 were considered as statistically significant.

## 3. Results 

A total of 18 stored skull flaps obtained during DHs between 2019 and 2020 were initially analyzed. After the exclusion of one outlier, 17 flaps remained for our analysis. Eight bone flaps were stored at −23 °C and nine at −80 °C. Nine patients (53%) were male. The median age of our cohort was 70 years; the median duration of cryopreservation was 10.5 months (2–17 months). The demographics of our cohort are shown in our previous publication [14]. 

The immunohistochemical staining was apparent in cell membrane and cytoplasm for PTH1R and OPG, respectively. The relative values (positive/all cells) per high power field (HPF) of PTH1 positive cells were comparable between our two subgroups, whereas OPG positive cells were more frequently observed in skull flaps stored at −23° according to our univariate analysis (PTH1R relative counts/HPF at −23 °C vs. at −80 °C, median: 1.61% vs. 2.34%, *p* = 0.923; OPG relative counts/HPF at −23 °C vs. at −80 °C, median: 6.91% vs. 1.32%, *p* = 0.039) (Table 1). After adjustment via the Poisson model for possible confounders, namely gender and age, OPG expression remained significantly higher in the group A.

The relative ratio of avital/total tissue surface was estimated in all specimens of both groups at two different periods of time, namely in December 2020 and June 2021. Surprisingly, a relevant degree of bone avitality was identified in all skull flaps, even after storage for several weeks. After subgroup analysis, increased values of avital tissue were observed in skull flaps stored at −23 °C vs. −80 °C at the initial evaluation but not at the repeated analysis after 6 months. Initial evaluation, avital tissue/total tissue surface at −23 °C vs. at −80 °C, median: 2.51% vs. 0.03%, *p* = 0.008; repeated evaluation, avital tissue/total tissue surface at −23 °C vs. at −80 °C, median: 13.16% vs. 8.34%, *p* = 0.470. It is noteworthy that the comparisons of repeated vs. initial measures at both –23 °C and –80 °C, identified significantly higher values of avital areas after 6 months (*p* < 0.001).

Our univariate analysis identified the prolonged storage of the skull flaps as a significant predictor of avital tissue (*p* < 0.001). Further epidemiologic and clinical factors, like age, sex, cause of malignant brain swelling (stroke vs. sTBI), additional skull fracture, infection prior to DH or the surgical duration of DH were not associated with the relative ratio of avital tissue. Our linear mixed regression model identified storage at −23 °C (*p* = 0.006) as well as longer storage times (*p* < 0.001) as independent prognostic factors for higher rates of bone avitality (Table 2). 

## 4. Discussion

Cranioplasty, although technically easily feasible, carries an extremely high rate of short term or secondary complications, the most severe of which are surgical site infection as well as aseptic bone necrosis with consequent bone autolysis [1,4,6,8,12,15,16]. Multiple and long-lasting surgeries, severe traumatic brain injury with fragmented skull flaps, younger age as well as comorbidities like hydrocephalus have been identified as prognosticators for secondary complications after cranioplasty [1,3,4,17].

Whether different patterns of skull flap storage may influence the course of the disease is disputable [14,18,19,20]. In this regard, we conducted our clinical trial DRKS00023283 [21], which aimed to clarify, among other things, whether storage at different degrees of °C, may prognosticate late complications after cranioplasty, such as infection and aseptic bone necrosis of the skull flap implants. In a first step, we performed sterile bacterial cultures as well as cultures after contamination with specific pathogens and showed similar microbiological behavior and infection potential of skull flaps stored at different temperatures [14]. In order to conclude our second endpoint, namely the involvement of patterns of storage in the course of aseptic bone necrosis, we carried out the present histopathological study.

Aseptic bone necrosis is observed only after cranioplasty with ABFs, a fact that results in higher overall complications after ABF rather than SF cranioplasty [6,8]. To this end, a relevant shift in the surgical management of the disease and the choice of material for cranioplasty has been already initiated; SFs are increasingly replacing ABFs for cranioplasties [5,6,13,22,23].

To date, we are aware that younger age [1,3,10,12,13], traumatic brain injury [4,17], size and thickness [24,25], reimplantation of multiple bone fragments [1,3,12,13] and surgical site infection [5] are well established prognostic factors for the development of aseptic bone necrosis [3,4,16,17]. The subsequent bone lysis may in turn initiate issues regarding cosmesis, protection, cranioplasty failure and reoperation as well as rare situations, like sinking skin syndrome [3,16,26]. Our current histological analysis aims particularly to answer the question whether the process of necrosis is already apparent and ongoing during the storage of the skull flaps and whether the temperature (−23 °C vs. −80 °C) and duration of storage may influence this procedure. To this end, we recognized unusually high rates of avital tissue areas in our explanted skull flaps compared to reference values for bone. Further, those skull flaps being stored for a longer period of time as well as those stored at −23 °C expressed significantly higher bone avitality. A repeated analysis of the same skull flap after 6 months showed even higher values of bone avitality.

The potential association of a longer duration of storage and reduced viability of a biological material has been discussed before [27]. Although, cryopreservation at −80 °C is in principle thought to preserve the viability and function of living cells and tissues for a long period of time, since cells enter into the phase of quiescence, a lethargic living state characterized by low metabolism [28,29], there are some research groups that report diminished viability of biological materials after longer durations of storage [16,18,27]. Sugimoto et al. analyzed the bone differentiation and proliferation capacities of 15 samples of human bone tissue-derived mesenchymal stromal cells after cryopreservation of 1 to 20 years and found an inverse correlation between the proportion of viable cells and the number of years of cryopreservation [27]. Bhaskar et al. conducted bone studies on explanted skull flaps supporting the notion that skull flaps stored at −30 °C for more than 6 months are not viable [18]. Chan et al. conducted an osteoblast culture on 18 skull flaps stored at –80 °C for 4 to 55 months and found no viable osteoblast population [30]. A similar osteoblast culture was performed on 47 skull flaps stored at −70 °C for 9 to 161 months without identification of viable osteoblast growth [31]. 

Whereas the possible relationship between a longer duration of storage and reduced bone viability has previously been speculated on, no associations between the temperature of storage and bone viability have been reported, yet. Our study provides novel robust data about the superiority of storage at −80 °C rather than −23 °C, which appears to be a more protective method of storage against the development of avital tissue, perhaps due to the entrance of the cell into quiescence. The design of our study, i.e., the direct comparison of storage at −23 °C vs. −80 °C for the first time at the same institution following the same management protocols (indications for DH and CP as well as surgical technique, timing of CP, preparation and remaining storage patterns of ABFs, etc.) allows for such far reaching conclusions, since it eliminates the selection bias which would be apparent if similar studies had been performed at various institutes at different locations. To date, we lack established protocols regarding the optimal storage of explanted skull flaps after DH [15,19]. ABFs are kept frozen at various temperatures mainly from −20 °C to −80 °C [19]. Our novel finding shows the clear benefit of storage at −80 °C and should be carefully considered for the future management and storage of ABFs.

PTH1R and OPG, two key players of bone metabolism, were additionally analyzed in our study. Increased PTH1R expression promotes the bone resorption rate via the activation of osteoclasts, whereas OPG protects the bone from resorption by binding to RANKL and TRAIL, thus inhibiting osteoclastogenesis [30]. The low levels of PTH1R and OPG observed in our study reflect a dramatically diminished but still present bone metabolism, as a consequence of cell freezing. In accordance with the notion that ABF cells stored at −80 °C demonstrate decreased metabolism, they also express lower OPG levels.

Synoptically, the current surgical management of cranioplasties includes implantation of both ABFs as well as SFs, according to each institute’s policy. Although, SFs are increasingly replacing ABFs, in particular in younger patients, after traumatic brain injury with multiple bone fragments, ABFs are still used mainly due to their lower costs [5,6]. A secure statement whether the reimplanted ABFs are viable or not is not possible. Even if some researchers failed to extract osteoblast from cultures of ABFs stored for an extremely long time [30,31,32,33], no data have yet been published for ABFs frozen only for several months. Furthermore, we cannot exclude that ABFs that appear non-viable at the time of their thaw, will undergo bone remodeling secondarily after their reimplantation and gain viability later. Our microscopical analysis did evaluate such ABFs, i.e., stored for several weeks or some months, and revealed relevant but not life restrictive modifications of the cell microarchitecture, as also supported by similar studies in fibular and rabbit models [34,35]. Further, these results are in line with the conclusions of Goettsche et al. [36]. They recently performed a histological analysis of osseous samples from aseptic bone necrosis of 11 ABFs, which had been stored after DH at −80 °C, reimplanted to the individuals and again explanted in the context of aseptic necrosis. The mean time between reimplantation of ABFs after DH and re-explantation of ABFs due to necrosis was more than 12 months, which left sufficient time for potential bone regeneration [36]. Although their microscopical investigation showed prominent structural changes, like loss of differentiation of cortical and cancellous bone, they still observed remodeling in terms of a build-up of vital bone tissue in some areas of bone. However, they also describe, in the tissue of the aseptic bone necrosis, high rates of avital osteocyte cavities, as we also do, and predominantly fibrotic and necrotic marrow spaces. Avital osteocytes are linked to local BRF, since the necrotic tissue recruits osteoclasts to engulf the apoptotic osteocytes and initiate bone autolysis [37,38,39,40].

Therefore, our study demonstrates that the reimplanted ABF may initially function as a biocompatible scaffold with minimal or no cell viability. However, the presence of adjacent healthy bone tissue could potentially stimulate bone regeneration (referred to as osteoconduction). The extensive presence of avital osteocytes though, which are inductors of osteoclast-regulated resorption, may act as source of bone regeneration failure and hinter graft incorporation leading to aseptic bone necrosis and bone lysis.

Our study is subject to various limitations, such as its retrospective character and the limited quantity of studied material, hindering a robust statistical analysis, which however are attributed to the nature of the disease. The present clinical trial, though, includes for the first time a direct unbiased comparison of different methods (temperature) of freezing for explanted skull flaps as well as an analysis of their bone metabolisms and bone cell architecture.

## 5. Conclusions

With regard to our new findings, we should think carefully before we reimplant ABFs that demonstrate high rates of avital areas. In our study, ABFs stored at −23 °C and for long periods of time >3 months, showed unusually high bone avitality, thus should preferentially not be used as material for cranioplasty and SFs should be implanted instead. Further, we should carefully reconsider whether reimplantations of ABFs in general are worth their high risk of aseptic bone necrosis with all their consequences, since according to our results avital tissue, even in smaller amounts, is already developed also in ABFs cryostored for only several weeks.

## Figures and Tables

**Table 1:** Expression profile of PTH1R and OPG as well as the ratio of bone avitality in both groups.

Variable	−23 °C	−80 °C	*p*-Value
	Median, Quartiles (25th–75th Percentiles)	Median, Quartiles (25th–75th Percentiles)	
PTH1R	1.61% (0.91–4.46)	2.34% (0.99–4.76)	*p* = 0.923
OPG	6.91% (3.88–12.53)	1.32% (0.99–2.44)	*p* = 0.039
Avital areas	2.51% (1.19–4.36)	0.03% (0.00–0.09)	*p* = 0.008
Avital areas (repeated assays after 6 months)	13.16% (6.86–16.68)	8.34% (5.22–15.36)	*p* = 0.470

**Table 2:** Results (fixed effects) from the log-linear mixed model with random intercept.

Variable	Regression Coefficients β (95% Confidence Interval)	exp(β) (95% Confidence Interval)	*p*-Value
Group (−23 °C vs. −80 °C)	2.77 (0.93; 4.60)	15.96 (2.53; 99.48)	0.006
Time (initial time point vs. 6 months later)	−4.26 (−6.09; −2.43)	0.01 (0.00; 0.09)	<0.001

## Data Availability

Supporting research data will be available upon request.

## Abbrevations

CP	Cranioplasty
DH	Decompressive hemicraniectomies
BFR	Bone flap resorption
DRKS	Deutsches Register Klinischer Studien (German Clinical Trials Register)
PTH1	Parathyroid hormone 1 receptor
OPG	Osteoprotegerin
H&E	Hematoxylin and Eosin
SF	Synthetic flap
ABF	Autologous bone flap
CT	Computer tomography
°C	Celsius
sTBI	Severe traumatic brain injury
ACM	Arteria cerebri media
FFPE	Formalin-fixed paraffin-embedded
HPF	High power field
RANKL	Receptor activator of nuclear factor kappa-B ligand
TRAIL	Tumor necrosis factor related apotosis-inducing ligand
